# A Point Mutation in *Phytochromobilin synthase* Alters the Circadian Clock and Photoperiodic Flowering of *Medicago truncatula*

**DOI:** 10.3390/plants11030239

**Published:** 2022-01-18

**Authors:** Soledad Perez-Santangelo, Nathanael Napier, Fran Robson, James L. Weller, Donna M. Bond, Richard C. Macknight

**Affiliations:** 1 Department of Biochemistry, University of Otago, Dunedin 9054, New Zealand; soledad.perezsantangelo@otago.ac.nz (S.P.-S.); Nathanael.Napier@plantandfood.co.nz (N.N.); donna.bond@otago.ac.nz (D.M.B.); 2 John Innes Centre, Norwich Research Park, Norwich NR4 7UH, UK; fran.robson@bristol.ac.uk; 3 School of Biological Sciences, University of Tasmania, Hobart 7001, Australia; jim.weller@utas.edu.au

**Keywords:** flowering time, phytochrome, circadian rhythm, legumes, *Medicago*

## Abstract

Plants use seasonal cues to initiate flowering at an appropriate time of year to ensure optimal reproductive success. The circadian clock integrates these daily and seasonal cues with internal cues to initiate flowering. The molecular pathways that control the sensitivity of flowering to photoperiods (daylengths) are well described in the model plant *Arabidopsis*. However, much less is known for crop species, such as legumes. Here, we performed a flowering time screen of a TILLING population of *Medicago truncatula* and found a line with late-flowering and altered light-sensing phenotypes. Using RNA sequencing, we identified a nonsense mutation in the *Phytochromobilin synthase* (*MtPΦBS*) gene, which encodes an enzyme that carries out the final step in the biosynthesis of the chromophore required for phytochrome (phy) activity. The analysis of the circadian clock in the *MtpΦbs* mutant revealed a shorter circadian period, which was shared with the *MtphyA* mutant. The *MtpΦbs* and *MtphyA* mutants showed downregulation of the *FT* floral regulators *MtFTa1* and *MtFTb1/b2* and a change in phase for morning and night core clock genes. Our findings show that phyA is necessary to synchronize the circadian clock and integration of light signalling to precisely control the timing of flowering.

## 1. Introduction

The control of flowering time involves mechanisms that integrate environmental cues, mainly light and temperature, to allow plants to flower when conditions are optimal for reproductive success. The regulation of flowering time requires complex pathways that have arisen through a relatively conserved set of genes in flowering plants [1]. The majority of flowering time control research has been undertaken in the model plant *Arabidopsis thaliana* (*Arabidopsis*) and has resulted in a comprehensive understanding of the molecular mechanisms involved [2]. Photoperiod, or daylength, is an environmental factor that has a major influence on the timing of flowering. In *Arabidopsis*, the perception of a long day (LD) is achieved through the regulation of the gene *CONSTANS* (*CO*) [3]. The circadian clock controls the transcription of *AtCO* mRNA, which peaks in the evening. The *AtCO* protein is stabilized by light through the activity of the photoreceptors crytochrome 2 (cry2) and phytochrome A (phyA) and promotes the expression of the flowering integrator *FLOWERING LOCUS T* (*FT*) and induction of floral identity genes to promote flowering [4].

The circadian clock, an internal timekeeping mechanism, has an integral role in photoperiod detection, and influences the seasonal control of a wide range of developmental traits, including flowering, shoot branching, stress tolerance, and the development of fruits and seeds [5,6]. The core circadian oscillator of *Arabidopsis* is composed of the morning genes *LATE ENOLGATED HYPOCOTYL (LHY)* and *CIRCADIAN CLOCK ASSOCIATED 1 (CCA1)*, the expression of which both peak at dawn. The *PSEUDO RESPONSE REGULATOR (PRR)* family of genes then peak from late morning to early evening, the latest being *PRR1* (also known as *TIMING OF CAB EXPRESSION 1 (TOC1)*), which is part of a direct negative feedback loop with LHY. Lastly, peaking in the evening are *GIGANTEA (GI)* and the evening complex genes *EARLY FLOWERING 3* and *4 (ELF3* and *ELF4)* and *LUX ARRHYTHMO (LUX)*, which all repress the genes expressed earlier in the day [7]. Understanding how flowering time mechanisms differ between *Arabidopsis* and other crop species is of key importance. 

*Medicago truncatula* (Medicago), commonly known as barrel medic, is a small annual legume that provides high-quality feed to livestock. It is part of the Fabaceae family that contains important crops such as lentil, pea, chickpea, alfalfa and clover. Medicago is a good model for the legume family because of its small diploid genome and short life cycle. Medicago has six *FT*-like genes, of which *MtFTa1* responds to long exposure to cold (vernalization) and is rapidly up-regulated in leaves in response to the LD photoperiod [8,9]. Integrator genes that regulate photoperiodic flowering genes have been discovered in Medicago [10,11,12]; however, it is still not clear which component/s is/are key for translating light inputs into the circadian clock to provide photoperiod-specific regulation of flowering. In *Pisum sativum* (pea), orthologs of the clock genes *ELF3, LUX, ELF4* all contribute to the repression of *FT* family gene expression and flowering under short days (SD) [13,14,15,16]. Unlike in *Arabidopsis*, the Medicago *CO*-like genes (*MtCOLa-MtCOLk*) do not appear to have a central role in the photoperiodic regulation of Medicago *FT*-like genes [17]. Furthermore, when the various *MtCOL* genes were overexpressed in the late flowering *Arabidopsis* *co* mutant, they were not able to rescue the flowering phenotype [17]. In addition, the *COLa* gene [18] is not misregulated in the pea evening complex mutants, despite substantial changes in *FT* expression, indicating that *PsCOLa* plays no role in flowering and photoperiod responsiveness in peas [13,19]. 

A legume-specific E1 protein has been proposed as a key integrator of photoperiodic flowering in SD legume soybeans, raising questions about its possible role in LD legumes such as Medicago. The soybean *GmE1* gene acts as a repressor of *GmFT* expression and flowering under LD conditions and has higher expression in LD conditions than in SD conditions [20]. *GmE1* has a diurnal transcriptional rhythm with a bimodal pattern, with peaks of transcription occurring four hours after dawn (ZT4) and just before dusk (ZT16) in LD conditions, and it is regulated by the soybean *PHYA* genes, *GmE3* and *E4*. When comparing the transcriptional profile of *GmFT*-like genes, the first transcription peak of *GmE1* overlaps with the transcription peaks of *GmFT2a* and *GmFT5a*, suggesting that *GmE1* might control expression of these *GmFT* genes [20]. Moreover, *GmE1* overexpression leads to late flowering plants with the repression of *GmFT2a* and *GmFT5a* and upregulation of the repressed *GmFTa1* and *GmFT4* genes [20,21,22,23,24]. In Medicago, the characterization of two *tnt1* mutants for *MtE1* showed a slight late-flowering phenotype under LD conditions, suggesting that *MtE1* might promote flowering through the up-regulation of *FT* genes [10,25]. However, when *MtE1* is overexpressed in wild-type (WT) soybean, *Arabidopsis* or rice, no effect on flowering time was observed [25]. Therefore, the putative role of *MtE1* in LD legumes still needs to be clarified.

It is clear that the perception of light is integral to plant flowering time control and photoperiod sensitivity and several plant photoreceptors have been shown to participate in this role. In *Arabidopsis*, both the red (R)/far-red (FR)-sensing phytochromes and the blue-sensing cryptochromes are known to influence CO stability and also adjust the alignment of the circadian clock to the day-night cycle. In legumes, *PHYA* genes appear particularly important as mutants in soybean are early flowering under LD and mutants in pea are late-flowering under LD [26,27]. A mutant of Medicago *phyA* (*MtphyA)* has recently been shown to cause late flowering under LD conditions and impair specific photomorphogenic responses [10].

The light-absorbing properties of higher plant phytochromes are derived from the presence of a covalently bound tetrapyrrole chromophore, phytochromobilin (PΦB) [28]. PΦB is synthesized in the plastid from 5-aminolevulinic acid (5-ALA) through two enzymatic steps; first, the conversion of heme to biliverdin IXα (BV) by a ferredoxin-dependent heme oxygenase (HO) and second, the reduction of BV to PΦB, which is achieved by the enzyme PΦB synthase (PΦBS) [28]. In *Arabidopsis* and pea, both HO mutants (known as *hy1* and *pcd1*, respectively) [29,30] and PΦBS mutants (known as *hy2* and *pcd2*, respectively) have been described [31,32]. These mutants share deficiency in their response to both R and FR light, which is manifested in multiple photomorphogenic defects, including reduced inhibition of hypocotyl elongation, pale appearance due to reduced chlorophyll content, and reduced expansion of cotyledons and leaves. However, they differ in their flowering phenotype as *Arabidopsis* *hy2* mutants are early flowering, while the pea *pcd2* mutant flowers late compared to WT [31,32]. This contrasting phenotype suggests that there is some difference in the flowering time pathway with respect to phytochrome action in the two species.

To expand our understanding of genes controlling flowering in legumes, we screened a TILLING population of *Medicago truncatula* for mutants displaying a late flowering phenotype. Here, we show that one such mutant is the result of disruption of the *MtPΦBS* gene, and in addition to delayed flowering and the characteristic photomorphogenic defects, this mutation shortens the period of circadian rhythms. We also compared these phenotypes with those of mutants for the *PHYA* and *PHYB* genes. Our data suggest that the late flowering of the *MtPΦBS* mutant reflects a deficiency of phyA and a delayed expression of *FT* genes that is also associated with altered circadian clock properties. These results support the idea that phyA inputs light information into the circadian clock, which is translated to regulate the expression of *MtFTb*1/2 to induce precise flowering of *Medicago truncatula* when grown under long days.

## 2. Results

### 2.1. Screening of a TILLING Population Reveals a Late-Flowering Mutant with Light-Sensing Deficiency

**Figure 1:**
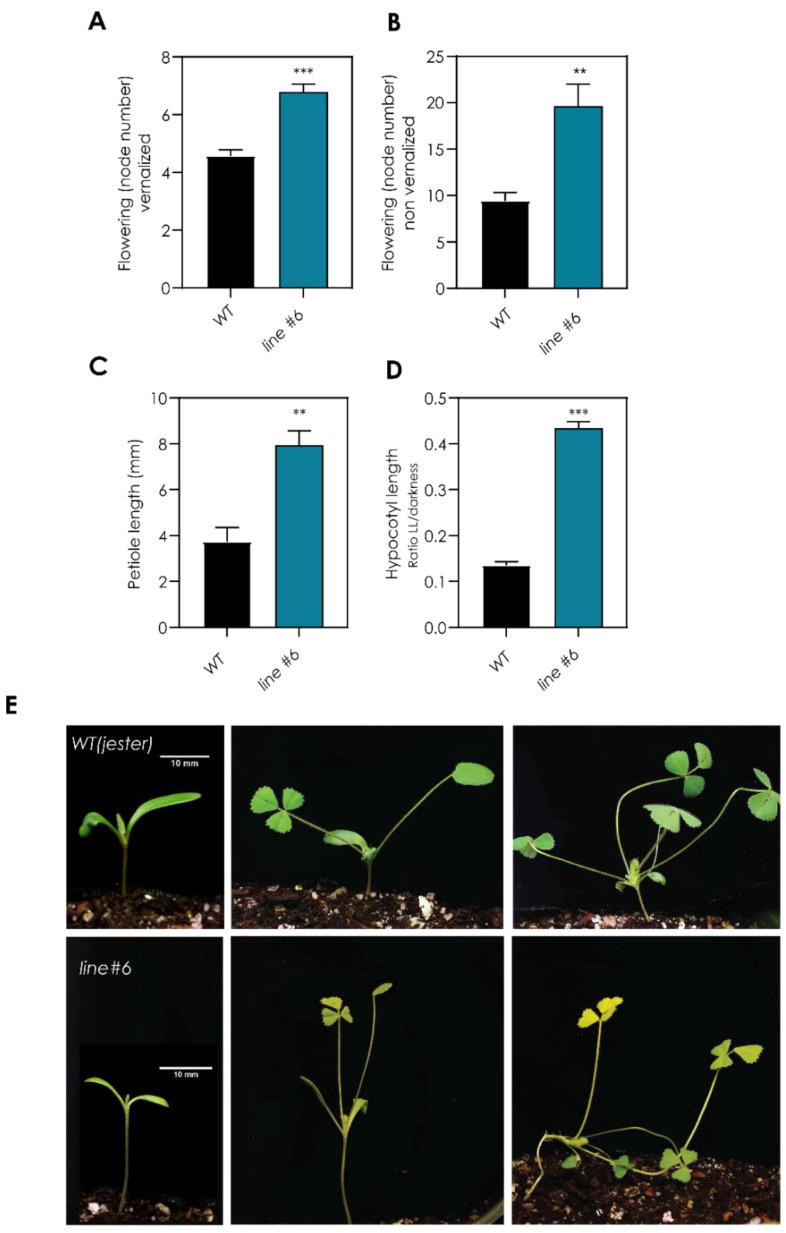
Characterisation of a late flowering mutant (*line#6*) with light-sensing deficiency. Flowering time of WT (Jester) and *line#6* of (**A**) vernalized plants (14d at 4 °C) under LD conditions and (**B**) non-vernalized plants under LD conditions. (**C**) Petiole length (mm) and (**D**) hypocotyl length (ratio LL/dark) under continuous light (LL). (**E**) Photograph of WT (Jester) and *line#6* plant at the cotyledon stage, after the expansion of the first trifoliate and after the expansion of the fourth trifoliate leaf produced from the apical meristem. Ruler = 10 mm. ** *p* < 0.01, *** *p* < 0.001 (Student *t*-test). Error bars represent SEM.

To identify key components in the integration of environmental signals to induce flowering in Medicago, we performed a forward screen of a Targeting-Induced Local Lesions IN Genomes (TILLING) population of *Medicago truncatula* plants at the John Innes Centre, Norwich, United Kingdom (“RevGenUK”) [33]. Thirteen putative late-flowering TILLING lines were grown under long-day (LD) conditions (16 h light/8 h darkness), and flowering time was measured as the number of nodes to the first flower. We focused on one line (*line#6*) which showed a strong late-flowering phenotype compared to wild-type plants in LD conditions (*line#6* = 20.6 ± 6.2 nodes, WT = 9.75 ± 1.0 nodes) (Figure S1). When germinated seeds were exposed to 4 °C for two weeks (vernalization) before being transferred to LD conditions, the mutant *line#6* flowered earlier than in LD but flowered later than the vernalized WT (*line#6* = 6.57 ± 0.78 nodes, WT = 4.60 ± 0.54 nodes, *p* < 0.001) (Figure 1A). An independent experiment confirmed the late flowering in LD conditions for the mutant *line#6* (Figure 1B). Furthermore, in LD conditions, *line#6* showed altered photo-morphogenesis, with longer petioles (Figure 1C), longer hypocotyl (Figure 1D) and smaller and paler leaves compared to WT plants (Figure 1E). The developmental phenotypes observed suggest that the mutant line has a mutation that is disrupting the light-sensing pathway which inputs into the photoperiodic sensing pathway, causing a late-flowering phenotype.

### 2.2. A Point Mutation in the MtPΦBS Gene Is Responsible for the Photo-Morphogenic and Late-Flowering Phenotypes

**Figure 2:**
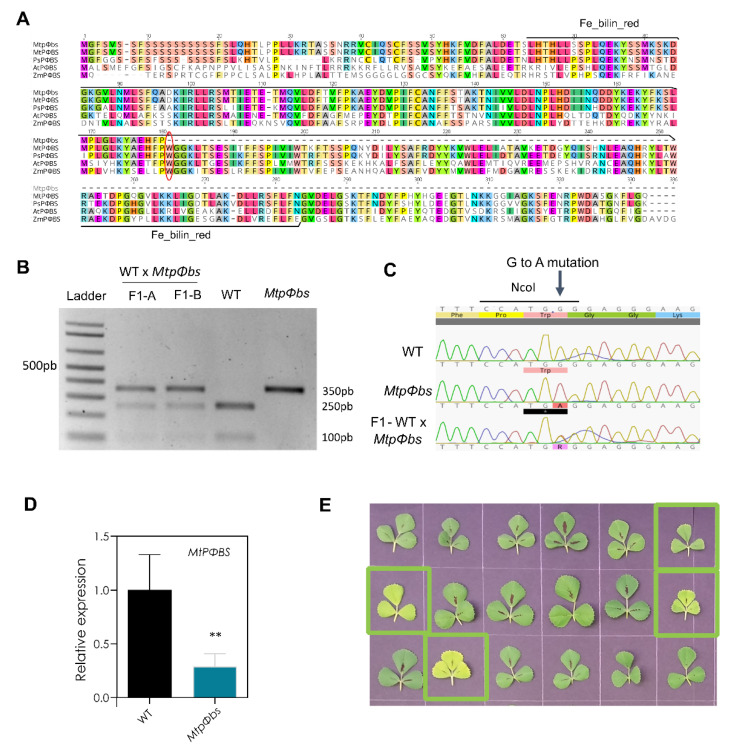
A point mutation in the *MtPφBS* gene is responsible for the photo-morphogenic and late-flowering phenotypes. (**A**) Alignment of Medicago Medtr5g097080 (*MtPφBS*) protein sequence with the PΦBS sequence from other plant species showing the Ferredoxin-dependent bilin reductase (Fe_bilin_red) domain (from 63aa to 280aa). Red oval shows the location of the stop codon found in the *Mtpφbs* mutant (178 aa *Mtpφbs* vs. 329 aa WT). (**B**) The G > A mutation in *Mtpφbs* causes a disruption of a NcoI restriction site. The NcoI restriction enzyme cuts PCR products from WT DNA (250 bp + 100 bp) but not *Mtpφbs* DNA (350 bp). Heterozygous F1 plants A and B results in all 3 PCR products (350pb + 250 bp + 100 bp), confirming the WT × *Mtpφbs* cross was successful. (**C**) Sequencing of the uncut products confirms the G > A change in the *Mtpφbs* mutant and a double (A/G) peak in F1 plants. (**D**) *MtPΦBS* expression levels were measured at ZT = 2 and primers were positioned upstream of the mutation site at the 5′ end of the gene on either side of the exon1/exon2 boundary. Relative expression of WT-normalized to housekeeper *MtPDF2*. ** *p* < 0.01 (Student *t*-test). Error bars represent SEM. (**E**) Picture of representative trifoliate leaves of F2-segregating population. Green squares indicate *Mtpφbs* mutants confirmed using the NcoI PCR assay.

To locate the mutation causal for the photo-morphogenic and late-flowering phenotype, high throughput RNA sequencing (RNA-seq) was performed using the mutant *line#6* and WT. The transcription profiles produced were used to discover potential SNPs and indels compared to the Medicago reference genome v4.2 [34] (Table S1). A potential flowering and light-sensing candidate gene list was generated from the literature on Medicago, soybean, pea and *Arabidopsis* (Table S2). We compared the RNA-seq reads against the reference genome to discover any genetic differences in the coding sequences of these candidate genes. The gene Medtr5g097080, with homology to *AtHY2*, was the only gene from the list of candidates to display a homozygous SNP variant (Figure S2A). The gene Medtr5g097080 is composed of nine exons with a coding sequence length of 990 bp (Figure S2B), and phylogenetic analysis suggests it is the Medicago *PΦBS* orthologue (*MtPΦBS*) (Figure S2C). Sanger sequencing confirmed a single nucleotide substitution (537 G > A) within the fifth exon of *MtPΦBS*, which causes an early stop codon (Trp179 > Stop). The conserved Ferredoxin-dependent bilin reductase (Fe_bilin_red) domain is present between 63aa and 280aa, and the eliminated region caused by the early stop codon is of high sequence conservation and likely functional importance (Figure 2A–C). *MtPΦBS* gene expression in *line#6* (now referred to as the *Mtpφb* mutant) was approximately 2-fold lower (Figure 2D), which could potentially reflect targeting of the mutant form to the nonsense-mediated decay [35].

To further clarify whether this substitution might be causal for the phenotypes observed, the *Mtpφbs* mutant was backcrossed to WT (Figure 2B–C) and co-segregation examined in the F2 generation. Seventy-five F2 plants grown under LD conditions were used to classify *Mtpφbs* mutants based on a ‘visual phenotype’, the observation of a collective set of unique mutant growth phenotypes (longer hypocotyl, longer petioles, and paler leaves) (Figure 2E and Figure S3).

Flowering time was analysed for forty vernalized F2 plants grown under LD and VLD conditions. We found that all the photo-morphogenic and the late-flowering phenotypes segregated with the putative causal SNP in the *MtPΦBS* gene (G > A; Trp197 > Stop) (Figure S3B–D). The SNP found in the *MtPΦBS* gene is the causal genomic change, given that it is tightly linked genetically and segregates with both flowering and photo-morphogenic phenotypes. We also checked genes with SNP variants within 2 MB of *MtPΦBS* gene in *line#6*, we found two genes: (1) a co-chaperone GrpE family protein, where the SNP did not cause a change in the amino acid sequences; (2) a nuclear protein Es2, with no literature indicating any plant orthologues could have a role in flowering and photomorphogenesis (Table S3).

In both *Arabidopsis* and pea, *pφbs* mutants result in phytochrome chromophore deficiency [31,32,36]. There are three groups of phytochromes, phytochromeA (phyA), phyB, and phyC, and each phytochrome has different roles. PhyA and phyB are the most abundant in *Arabidopsis*, and they play dominant roles in photo-morphogenic responses [37]. Therefore, we next analysed the photo-morphogenic phenotypes, petiole and hypocotyl elongation under different light conditions for *MtphyA, MtpΦbs* mutants, a newly isolated *MtphyB* mutant. The *MtphyB* mutant was derived from a TILLING population (in the A17 background) treated with EMS that resulted in a line with a change of 1773 G > A (protein sequence change Trp547 > Stop) in the *PHYB* gene (Medtr2g034040) (Figure S4A). *MtPHYA* expression is reduced 2-fold in the *MtphyA* mutant (Figure S4B) and *MtPHYB* expression is reduced 5-fold in the *MtphyB* mutant (Figure S4C). Phytochromes have a broad spectrum of light absorption, where phyA is considered to be the main far red (FR) sensor and phyB a dominant regulator of red (R) response. Under constant white light (LL) conditions, we observed that both *MtphyB* and *Mtpφbs* mutants had a longer hypocotyl compared to their respective WTs ((Figure S5A). In contrast, for FR conditions, we observed a longer hypocotyl phenotype for *MtphyA*, as previously reported [10], and for *Mtpφbs* (Figure S5B). Lastly, under LD conditions, we observed a clear longer petiole for both *MtphyB* and *Mtpφbs* mutants and to a less extent for *MtphyA* (Figure S5C). With these data, we can say that the photo-morphogenic phenotypes observed for *Mtpφbs* mutants are likely due to the loss of function of both phyA and phyB phytochromes.

### 2.3. MtE1, a Legume Specific Flowering Gene, Is Only Expressed in LD Conditions and It Is Downregulated in the Mtpφbs Mutant

**Figure 3:**
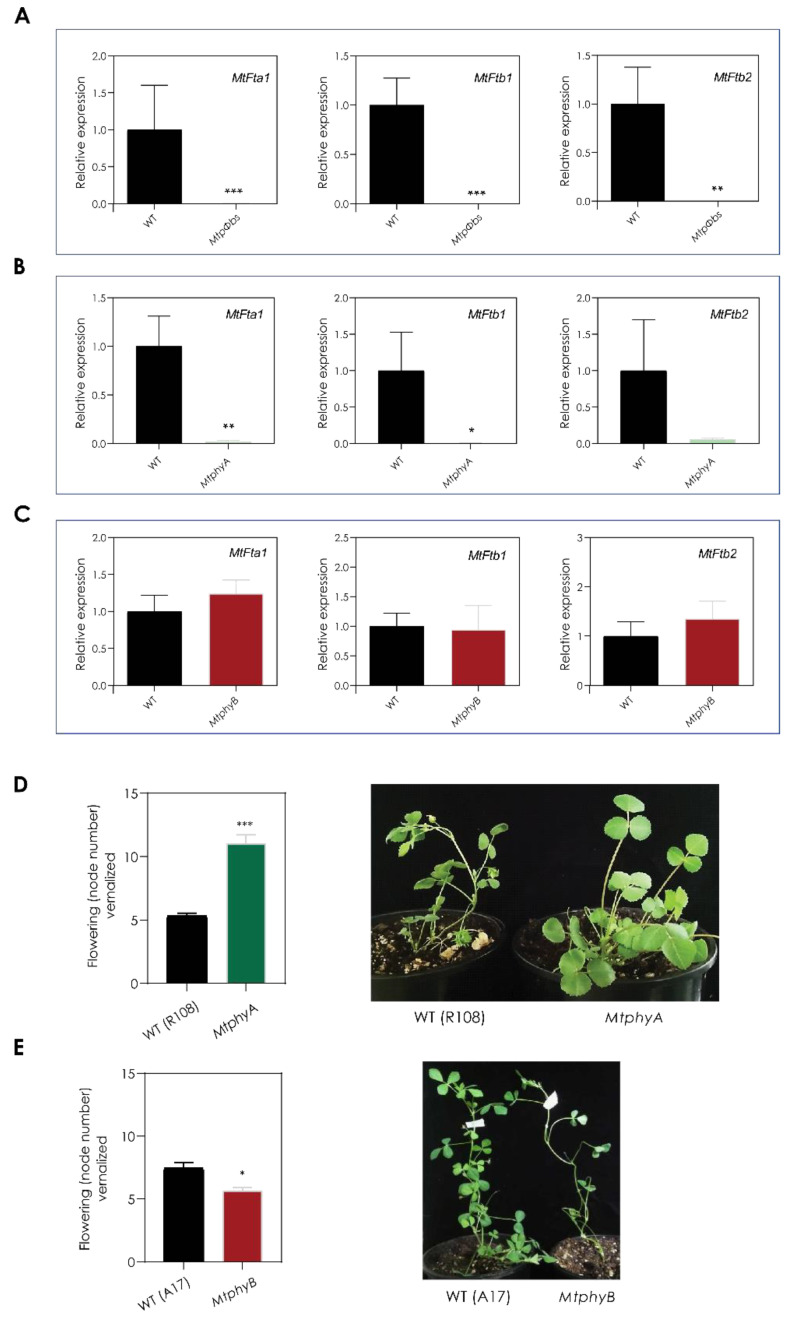
The *MtpΦbs* mutant shows downregulation of *MtFT*-like genes. Gene expression of *Mt, FTa1, FTb1 and MtFTb2* in (**A**) *Mtpφbs*, (**B**) *MtphyA* and (**C**) *MtphyB* mutants, and respective WT in VLD conditions. Flowering time was evaluated as number of nodes to first flower and photographs were taken at the time the first flower emerged for (**D**) *MtphyA* and (**E**) *MtphyB* and respective WT in VLD conditions. Relative transcript abundance was measured in the fully expanded trifoliate leaves of 14-day-old plants. Tissues were harvested 2 h after dawn in VLD conditions. Relative gene expression was measured by qRT-PCR with normalization to *MtPDF2*. Data are the mean ± SEM of three biological replicates and relative to the highest WT value. VLD = Vernalized long-day. *** *p* < 0.001; ** *p* < 0.01, * *p* < 0.05 (Student *t*-test).

To deepen our understanding of the molecular mechanisms by which the mutation in *MtPΦBS* alters flowering time, we first analysed the expression of known promotors of flowering—the *FT*-like genes: *MtFTa1*, *MtFTb1* and *MtFTb2* [8]. For this, plants were vernalized and grown in LD conditions (VLD) and the second trifoliate leaf was harvested at ZT2, 2 h after dawn, when expression is expected. We observed that *MtFTa1*, *MtFTb1* and *MtFTb2* genes were completely downregulated in *Mtpφbs* mutants (Figure 3A). This result is consistent with the late flowering time phenotype observed for *Mtpφbs* mutants. As expected, we also observed a complete downregulation of the three *MtFT* genes in the late-flowering *MtphyA* mutant [10] (Figure 3B) but not in the *MtphyB* mutant (Figure 3C).

When we analysed the flowering phenotypes of the *Mtphy* mutants, we found that *MtphyA* plants were late flowering compared to WT plants (*MtphyA* = 11 ± 1.4 nodes, WT = 5.3 ± 0.5 nodes under VLD conditions) (Figure 3D), which is consistent with recently published data [10], whereas a slight early flowering phenotype was observed for *MtphyB* when compared to WT plants *(MtphyB =* 5.7 ± 0.8 nodes, WT = 7.4 ± 1.3 nodes under VLD conditions) (Figure 3E). These results suggest that the late-flowering phenotype of *MtpΦbs* is a result of a loss of the function of *PHYTOCHROME* *A* (*MtPHYA*) and not *MtPHYB* under white-light VLD conditions.

**Figure 4:**
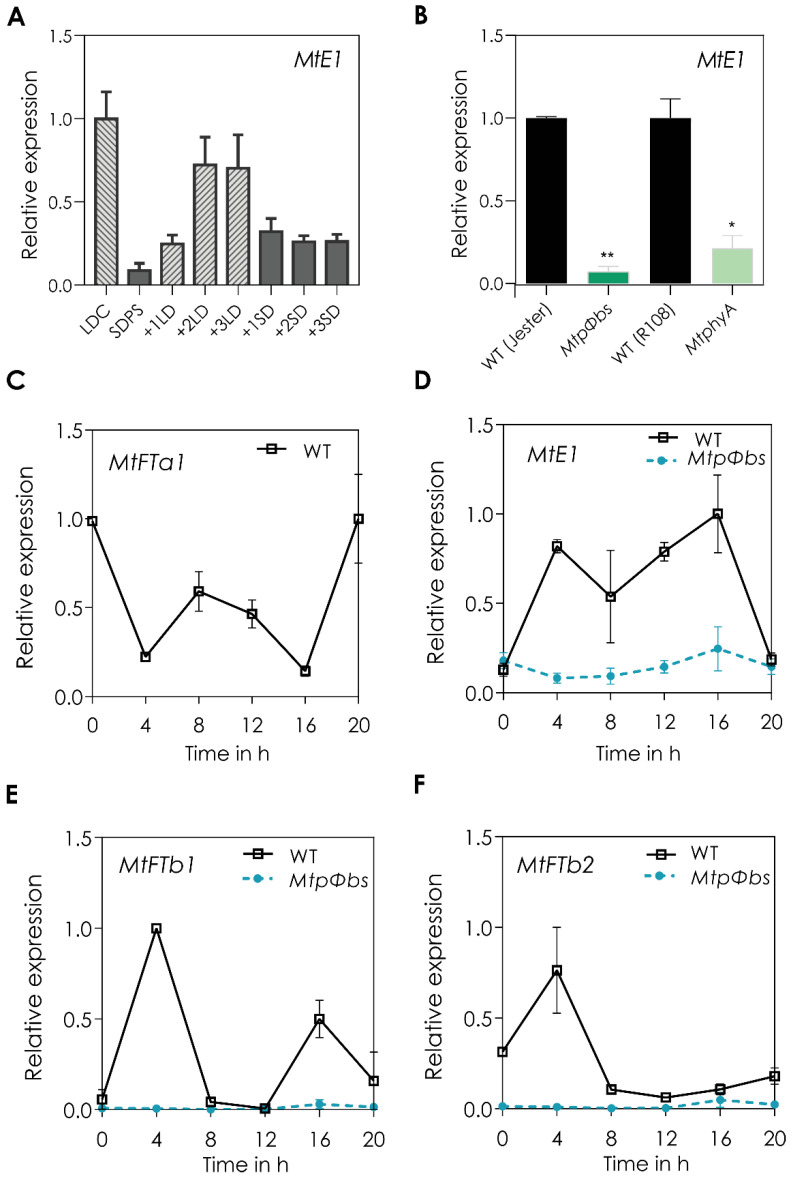
The legume-specific flowering gene, *MtE1*, is downregulated in the *Mtpφbs* mutant. (**A**) Response to changes in photoperiod. WT R108 leaves were collected at ZT4 of plants grown in LD and SD conditions. Plants grown in SD were then transferred to LD conditions for 1, 2, and 3 days before being transferred again to SD for 3 more days. *MtE1* gene expression in (**B**) *MtpΦbs* and *MtphyA* mutant, and respective WT. Relative transcript abundance was measured 4h after dawn in LD conditions. Diurnal expression profiles of (**C**) *MtFTa1*, (**D**) *MtE1*, (**E**) *MtFTb1* and (**F**) *MtFTb2*. Plants were entrained for 21 days under LD conditions. Tissues were harvested every 4 h for a 24 h period. Relative gene expression was measured by qRT-PCR with normalization to *MtPDF2*. Data are the mean ± SEM and relative to the highest WT value. SD = short day (8 h light/16 h dark), LD = long-day (16 h light/8 h dark). ** *p* < 0.01, * *p* < 0.05 (Student *t*-test).

The photoperiod pathway of flowering time involves the integration of light signals and specific expression of genes at a particular time of day/season. With the absence of a functional CO in legumes to integrate the photoperiod signals to regulate expression of the *FT* genes, *MtE1*—a legume-specific flowering gene—could be a good candidate for this role. To first analyse whether *MtE1* is genuinely responsive to day length, the expression of this gene was examined in response to changes in photoperiod. RNA was obtained from Medicago R108 leaves collected at ZT4 after plants were grown in SD and then transferred to LD conditions for 1, 2, and 3 d. Expression of *MtE1* in pre-shift SD conditions began at low levels and was upregulated when the plant was transitioned into LD, and its expression increased further over the 3 LDs (Figure 4A) [38]. This data shows that *MtE1* is induced in LD conditions and responds to changes in photoperiod, confirming what was documented by Jaudal et al. [10].

Next, we analysed the expression patterns of *MtE1* in the late-flowering *MtpΦbs* and *MtphyA* mutants. For this, plants were non-vernalized and grown in LD conditions, and the second trifoliate leaf was harvested at ZT4, 4 h after dawn where peak expression of GmE1 is observed [23]. We found a strong downregulation of *MtE1* in the *MtPΦBS* mutant and in the *MtphyA* mutant (Figure 4B) [10].

The diurnal patterns of *MtE1* and *MtFT* genes was analysed in WT and *MtpΦbs* mutant backgrounds by growing plants in LD and taking samples every 4 h for a 24 h period. In WT plants we observed a dual peak of expression at dawn (ZT0) and mid-afternoon (ZT8) for *MtFTa1* (Figure 4C), we observed two clear peaks at ZT4 and ZT16 for *MtE1 and MtFTb1* (Figure 4D,E), and a single peak at ZT4 for *MtFTb2* (Figure 4F). In contrast, we observed a complete deregulation of all four genes in the *MtpΦbs* mutant background. This revealed that *MtpΦbs* late-flowering phenotypes are caused by a deregulation of *MtFTa1*, *MtFTb1* and *MtFTb2* expression. Given that *MtE1* and *MtFTb1* are induced by LDs and have a similar expression pattern, both peaking at ZT4 and ZT16, it is possible that they are regulated by a common mechanism requiring active phyA. As the peak of *MtE1* and *MtFTb1* expression occurs about 4 h before the peak expression of *MtFTa1* (providing time for their mRNA to be translated), one or both these genes might be involved in the transcriptional regulation *MtFTa1.* Future experiments examining the expression of the *MtFT* genes in *MtE1* and *MtFTb1* mutant or overexpression lines will be required to investigate this possibility.

### 2.4. The MtpΦbs Mutant Has Altered Circadian Clock Function

**Figure 5:**
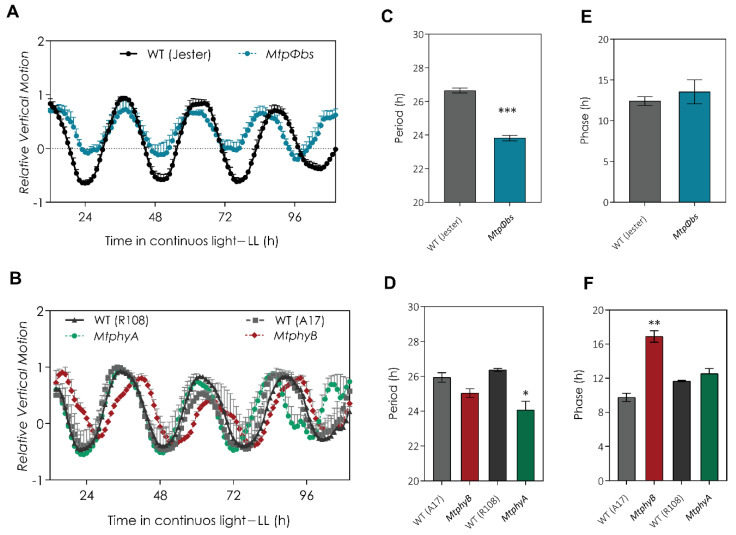
The *MtpΦbs* mutant has an altered circadian clock, and it is shared with the *MtphyA* mutant. Circadian rhythm of cotyledon movement was recorded in plants entrained under long-day conditions (16 h light/8 h darkness) and then transferred to constant light (LL) for 5 days. Relative vertical motion was obtained for (**A**) WT (Jester); black circles, *MtpΦbs* mutant blue circles; (**B**) WT (R108); black triangle; *MtphyA* green circles; WT (A17) grey squares and *MtphyB*, red circles. *n* = 4 for all genotypes. (**C**,**D**) Period length of cotyledon movement estimated by fast Fourier transform– nonlinear least test (FFT-NLLS). (**E**,**F**) Phase of cotyledon movement. Error bars represent SEM. * *p* < 0.05, ** *p* < 0.01, *** *p* < 0.001 (Student *t*-test). h = hours.

Since phyA could be integrating light information to the circadian clock and translating to trigger flowering, we decided to study whether the *Mtpφbs*, *MtphyA* and *MtphyB* mutants had changes in their regulation of circadian rhythms. For this, we measured the cotyledon movement, which is a robust physiological output of the clock. Plants are entrained in LD and circadian period and phase were measured in continuous light (LL) conditions where the cotyledon movement is only controlled by the circadian clock. For the *Mtpφbs* mutant, we observed an altered circadian phenotype, with an approximately 2 h shorter period compared to WT plants (23.82 ± 0.17 h and 26.65 ± 0.15 h, respectively, *p* < 0.001), but no difference in phase. (Figure 5A,C,E). When we measured the cotyledon movement for *MtphyA* mutant, we observed an altered circadian phenotype with an approximately 2 h shorter period compared to WT plants (*MtphyA* = 23.12 ± 0.5 h and WT = 26.28 ± 0.1 h, respectively, *p* < 0.05) (Figure 5B,D,F). No differences were observed for the period between *MtphyB* and WT plants (Figure 5B,D). However, a significant shift in the phase of cotyledon movement was observed for *MtphyB* compared to WT plants (*MtphyB* = 16.9 ± 0.67 h and WT = 9.7 ± 0.47 h, respectively, *p* < 0.01) (Figure 5B,F). These data suggest that the circadian period phenotype of the *Mtpφbs* in continuous white light is partially due a loss of function of phyA.

### 2.5. The MtpΦbs Mutant Has a Shift in Expression of the Core Clock Genes

**Figure 6:**
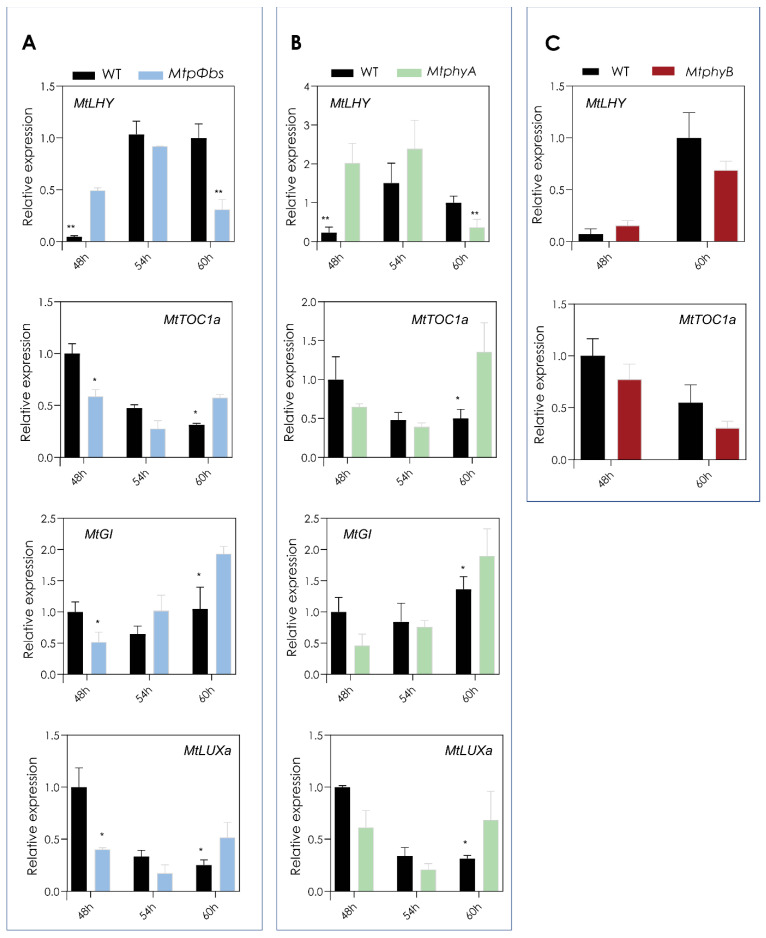
The *MtpΦbs* mutant has a shift in expression of the core clock genes. Gene expression of core clock genes in (**A**) *Mtpφbs*, (**B**) *MtphyA* and (**C**) *MtphyB* mutants, and respective WT in LL3. Relative transcript abundance was measured in the fully expanded trifoliate leaves of 24-day-old plants. Tissues were harvested every 6 h after dawn of the third day in LL after being entrained in LD for 21 days. Relative gene expression was measured by qRT-PCR with normalization to *MtPDF2*. Data are the mean ± SEM of three biological replicates and relative to the highest WT value. ** *p* < 0.01, * *p* < 0.05 (Student *t*-test). LD = long-day (16 h light/8 h dark). LL3 = third day of free-running conditions (constant light).

To further understand how the mutation in the *Mtpφbs* gene affects the regulation of the circadian clock, we analysed the expression pattern of the Medicago core clock genes under diurnal (LD conditions). For this, plants were grown in LD conditions and samples were taken every 4h starting at ZT = 0 (sunrise) for a day. We observed that the diurnal expression patterns were maintained with a small downregulation of expression at ZT4 and ZT12 for the morning and afternoon core clock genes, *MtLHY* and *MtLUXa*, respectively (Figure S6A–D). This was also observed for *MtLHY* in the *MtphyA* mutant, but not the *MtphyB* mutant (Figure S6E,F). Next, we specifically studied the control of the circadian clock on gene expression patterns. For this, we grew plants in LD conditions before transferring them to LL (free-running conditions), where gene expression is only controlled by the circadian clock and not by the dark/light cycles. Interestingly, we observed a strong change in the expression profiles of the morning gene *MtLHY*, the night gene *MtTOC1a* and evening genes *MtGI* and *MtLUXa* in the *Mtpφbs* mutant background (Figure S7). When we compared the core clock expression profiles in free-running conditions with the *MtphyA* and *MtphyB* mutants, we observed the same expression patterns as *Mtpφbs* for *MtphyA* (Figure 6A,B), while *MtphyB* did not have any changes in expression compared to wild-type plants (Figure 6C). This is consistent with the same short circadian period of cotyledon movement observed for the *Mtpφbs* and *MtphyA* mutants. When we investigated the expression of *MtPHYA* in the *Mtpφbs* background under diurnal and free-running conditions, we observed an earlier peak of expression compared to WT (Figure S8). Together, these results suggest that the circadian phenotype of *Mtpφbs* is due to mis-function of phyA.

## 3. Discussion

Photoperiodic control of flowering in *Arabidopsis* has been studied in detail [2], but increasing evidence suggests that this mechanism differs in other plant species, such as the legume Medicago [8,9]. The *CONSTANS* gene has a well-established central role in the photoperiod sensing pathway in *Arabidopsis*, but this was not observed for Medicago *MtCOL* genes [17]. In contrast, the role of *FT* genes as key regulators of flowering time is shared between *Arabidopsis* and Medicago [8]. To further our understanding of the control of flowering time in legumes, we screened a *TILLING* population of Medicago plants for flowering time behaviour and identified a late flowering line with altered photo-morphogenesis (Figure 1 and Figure S1). We discovered a homozygous nucleotide substitution within *MtPΦBS*, the Medicago orthologue of the *Arabidopsis* *HY2* gene. This was a G > A base substitution at the 537th base of the coding sequence, within the fifth exon of the *MtPΦBS* genomic region, which causes an early stop codon (537 G > A; Trp179 > Stop) (Figure 2 and Figure S2). The *PΦBS* enzyme has been shown to function within the phytochrome chromophore biosynthesis pathway and is responsible for converting Biliverdin IX to phytochromobilin [39]. Consistent with both the *Arabidopsis* and pea *pφbs* mutants, Medicago plants defective in *PΦBS* have elongated hypocotyls and petioles, a phenotype shared with both *MtphyA* and *MtphyB* mutants (Figure S5). As only a single *MtpΦbs* mutant was found, complementation would be needed to prove the mutation is causal. However, the correlation between the *MtpΦbs* mutation and the photomorphogenic phenotypes, hypocotyl and petiole length, flowering time, and leaf colour in the F2 population provides strong evidence (Figure 2E and Figure S3). Moreover, the *Mtpφbs* mutant shares a late-flowering phenotype with pea *PspΦbs* mutants [31]. Interestingly, *Arabidopsis* plants defective in *AtPΦBS* showed an early flowering behaviour [40]. This difference in flowering time phenotype between *Arabidopsis* plants defective in *PΦBS* compared to pea and Medicago counterparts may be related to the complex regulation and stabilization of CO in *Arabidopsis* and a lack of function of this protein in legumes. The mechanism to restrict *AtCO* expression to the evenings of LDs in *Arabidopsis* is intricate and includes the clock genes *AtGI* and *AtFKF1*, the phytochromes phyA and phyB, the cryptochromes cry1 and cry2 and the *AtCDFs* [2,4]. Therefore, with an absence of CO, phytochromes may function differently in the legume *pφbs*-deficient mutants, which could explain the difference observed in the flowering phenotype between *Arabidopsis* and legumes. The photomorphogenic phenotypes we observed with the *Mtpφbs* mutant suggest that *MtPΦBS* affects flowering time via the photoperiod pathway. Consistent with this, we have shown here that *Mtpφbs* mutant plants can still respond to vernalization (Figure 1A), a result previously unknown for *PΦBS*-defective legumes, as peas do not have a strong vernalization response [29,31].

In soybean, *GmE1* has been shown to be a negative regulator of flowering, inhibiting flowering in LD conditions. It has been proposed that the presence of *GmE1* explains how this short-day plant (SDP) can respond differently to daylength compared to other LD legumes [41]. The difference in the mechanism of integration of light signalling between long-day plant (LDP) and SDP that explains how the switch in flowering time can occur is not known. In Medicago, a *tnt1* line that disrupts *MtE1* had a subtle late-flowering phenotype [25]. Consistent with these data, we observed a strong downregulation of *MtE1* in the *Mtpφbs* late-flowering mutant, suggesting that *MtE1* could be involved in the activation of flowering under LD conditions. Further, when the diurnal patterns of *MtE1* were analysed, two peaks of expression, at ZT4 and ZT16, were observed, with no expression over 24 h for the *MtpΦbs* mutant (Figure 4). This diurnal pattern was also observed for *GmE1* [23]. The role of *MtE1* as a positive regulator of flowering time in Medicago needs to be confirmed, and it is still unknown if a CO-like protein acts within the same pathway as *MtE1* [17].

Light input to the circadian clock is a complex feedback system, where light input to the central oscillator acts as a feedback mechanism to control phytochrome expression in a day. The mechanisms from light perception to clock function are not well understood in LD legumes. In *Arabidopsis*, a *phyA* mutant shows a longer circadian period in red light [42] and flowered significantly later than the wild type in continuous R+FR light, which indicated that phyA promotes flowering in FR conditions [43]. In pea, a dominant mutation in the *PsPHYA* gene results in early photoperiod-independent flowering [44]. We found the *Mtpφbs* and *MtphyA* mutants had a shorter circadian period in continuous white light (Figure 5), and the analysis of core clock genes showed a shared change in their expression pattern for both *Mtpφbs* and *MtphyA* mutants (Figure 6 and Figure S6). This suggests that the clock phenotype of the *Mtpφbs* mutant is at least partially due to the mis-function of phyA. The shared flowering and circadian phenotypes for *MtpΦb* and *MtphyA* suggest an important role of phyA inputting light information into the clock to induce photoperiodic flowering.

Lastly, the *MtphyB* mutant showed a characteristic longer hypocotyl under white light (shared with *MtpΦbs* mutant) but no difference in FR light (Figure S5), as seen in the *Arabidopsis* *phyB* mutant and the pea *lv* mutant deficient in phytochrome B [45,46]. As the *AtphyB* and *pea lv* mutants also have a longer hypocotyl under R light, it is expected *MtphyB* mutant will have the same response, but this will need to be confirmed.

## 4. Materials and Methods

### 4.1. Plant Materials

*Medicago truncatula* Jemalong A17, Jester, *MtphyA, MtphyB* and R108 were used as described. The *Mtpφbs* line was derived from a forward phenotype screen of an A17 EMS-mutagenized TILLING population (RevGenUK, Norwich UK https://www.jic.ac.uk/research-impact/technology-research-platforms/reverse-genetics/, accessed on 6 December 2021) [33] and contains a point mutation G537A (W197X) in the *MtPΦBS* gene (Medtr5g097080). The *MtphyA* mutant comes from a *Tnt1* retrotransposon insertion mutant population and has an insertion in the *PHYA* gene (Medtr1g085160) in the R108 background (line NF1583) [10,47]. The *MtphyB* mutant was derived from a TILLING population (in the A17 background) treated with EMS that resulted in a line with a change 1773 G > A (protein sequence change Trp547 > stop) in the *PHYB* gene (Medtr2g034040).

### 4.2. Growth Conditions

Scarified seeds (seed surface scratched using fine sandpaper) were water imbibed and germinated at room temperature on wetted filter paper in a Petri dish in the dark. When radicles extended to a sufficient length (~10 mm), the seedlings were transferred into growth media (3 parts soil (Yates Professional Potting Mix, Auckland, New Zealand) and 1 part vermiculite) in controlled environment rooms. Plants were grown in a growth cabinet supplied with white light (TL5 28W low-pressure mercury discharge lamp, Philips, Auckland, New Zealand) ~90–110 µmol/m^−2^s^−1^) in 65% relative humidity at 25 °C or in a growth room, supplied with white light (~80–100 µmol/m^−2^s^−1^) in ~60% relative humidity at 18–23 °C. For vernalized conditioning, Medicago seeds were scarified and water imbibed overnight in the dark at room temperature before being placed at 4 °C in the dark for two weeks on wetted filter paper in a Petri dish. The seedlings were then planted into soil and put into the growth cabinet under the specified light conditions.

### 4.3. Hypocotyl Length

For the hypocotyl length assay, seedlings were arranged in a row on square agar plates (12 × 12 cm), sealed with micropore tape and propped vertically. The agar, Becton-Dickinson Bacto-Agar 214010 (Mount Pritchard, Sydney, Australia), was used at 0.8% (*w*/*v*) concentration in water. The plates were placed in the growth room set at different light conditions (~1 µmol/m^−2^s^−1^) continuous white light or Far Red (FR) light, while the dark experiments were conducted in the same conditions, but the plates were wrapped in aluminium foil.

### 4.4. Flowering Time Analysis

For flowering time analysis, plants were grown under long days (16 h light/8 h dark) at a constant temperature of 22 °C. Flowering time was estimated by counting the number of nodes produced until the appearance of the first flower. The node that contains the flower was included in the measurement, but the node of the unifoliate leaf was not.

### 4.5. Circadian Leaf Movement Analysis

For leaf movement analysis, seeds were sown into small circular pots (filled with growth media) and entrained in LD (16 h light/8 h dark) conditions at 22 °C for three days until cotyledons were fully expanded. Then, plants were transferred to continuous light (LL) and constant temperature (22 °C) conditions. Photos were taken every hour for six days using Wingscapes TimelapseCam Cameras and analysed by recording the plants’ vertical leaf motion (relative vertical motion: RLM) with a program developed in java [48] based on Matlab code from the software TRiP: Tracking Rhythms in Plants (https://github.com/KTgreenham/TRiP; accessed on 6 December 2021) [49]. The circadian period was obtained via fast Fourier transform nonlinear least-squares (FFT-NLLS) analysis using the online program BioDare2 (www.biodare2.ed.ac.uk; accessed on 6 December 2021) [50]. The first 24 h were excluded from the analysis to remove potential noise caused by the transfer from entrainment conditions to constant conditions.

### 4.6. Genotyping and DNA Sequencing, Protein Alignments and Phylogenetic Analysis

PCR products were sequenced via Sanger sequencing using a capillary ABI 3730xl DNA Analyser (Applied Biosystems) at Genetic Analysis Services (GAS), Department of Anatomy, University of Otago (New Zealand). Geneious^®^ (Geneious Pro software, Biomatters Ltd., Auckland, New Zealand) version 10.2.3 was used to analyse sequencing data and investigate/compare genome sequences between species to carry out sequence analysis. For analyses involving the Medicago genome, the latest version of the *Medicago truncatula* genome (Medtr 4.0v2) with annotations was used as a reference [34]. Protein alignment was performed using Geneious^®^ MUSCLE Alignment with default settings and the domain search was performed in http://www.ebi.ac.uk/interpro/ (accessed on 6 December 2021) Fe_bilin_red domain (from 63aa to 280aa).

### 4.7. Generation of the F2 Population

The SNP found in *MtPΦBS* disrupted a NcoI restriction site and was used to confirm the F1 cross was successful. The F1 plants were then self-pollinated to generate an F2 population that was used to investigate whether the late-flowering phenotype segregated with the mutation in the *MtPΦBS* gene.

### 4.8. RNA Extraction and RNA Sequencing

The fifth trifoliate leaf of 3 individual WT and 3 individual *line#*6 plants were harvested in a 1.5 mL microcentrifuge tube containing two 3.2 mm diameter steel beads (Lab Supply Ltd., Dunedin, New Zealand) and homogenized to a fine powder using the Qiagen TissueLyser II. RNA was extracted using the Plant RNA purification reagent (Invitrogen™ Thermo Fisher Scientific™, Auckland, New Zealand) following the manufacturer’s instructions.

Six RNAseq libraries were prepared using NEXTflex^®^ Rapid Directional qRNA-SeqTM Kit (Bio-Scientific, Kirrawee, Australia) as instructed by the manufacturer’s protocol. The same amount of each RNAseq library was pooled and run on one lane of a 125PE HiSeq2000 (Illumina, Singapore).

### 4.9. Processing of RNA Sequencing Reads

FastQC was used to analyse the quality of the reads (Andrews 2010). Trimmomatic v0.36 [51] was used to trim adapter and barcode sequences (minimum length of 39 bp with headcrop = 9). Sequences were aligned to the Medicago Medtr 4.0v2 reference genome [34] via TopHat2 v2.1.1 with default parameters and -library-type first strand, -GTF, and -b2-sensitive (v2.3.3 of bowtie2 [52]). Integrative Genomics Viewer [53] (IGV v.2.4.3) was used to view the RNA-seq reads against the reference genome.

The raw sequencing data have been uploaded to the BioProject collection and are available under accession number PRJNA794171 [54].

### 4.10. cDNA Synthesis and Quantitative RT-PCR

Total RNA (1 μg) was used in a gDNA Eraser (TaKaRa) reaction following the manufacturer’s instructions, to remove any contaminating gDNA. Genomic DNA-free RNA was then used to synthesize first-strand cDNA with a PrimeScript RT reagent kit (TaKaRa, San Jose, CA, USA).

Synthesized cDNA (diluted 1 in 30) was used to analyse the expression of genes using real-time quantitative RT-PCR (qPCR). The Medicago *TUBULIN BETA-1 CHAIN* (*MtTUB:* Medtr7g089120) or Medicago *SERINE/THREONINE-PROTEIN PHOSPHATASE 2A* (*PP2A*; also known as *PROTODERMAL FACTOR 2*; *MtPDF2*: Medtr6g084690) genes were used as the internal controls [55]. cDNA was amplified with SYBR FAST qPCR master mix (Kapa Biosystems, Dunedin, New Zealand) using the LightCycler 480 (Roche, Auckland, New Zealand). All reactions were carried out following the manufacturer’s protocol, and negative controls for each primer pair were included by replacing the template with nuclease-free ddH2O. The cycling conditions were as follows: initial denaturation at 95 °C for five minutes, followed by 50 cycles of denaturation at 95 °C for 10 s, primer annealing at 58 °C for five seconds, and extension at 72 °C for eight seconds. Relative gene expression levels were calculated using the 2(−delta delta C(T)) method [56]. Primers used for qRT-PCR experiments can be found in Table S4.

## 5. Conclusions

In conclusion, the *MtpΦbs* mutant likely affects flowering by disrupting the ability of phytochromes (mainly phyA) to perceive light and integrate light signals through the circadian clock to regulate flowering in a timely manner. Therefore, the expression of many other flowering-time genes is probably altered, making this mutant a powerful tool to identify additional downstream genes involved in flowering time control. The results presented here have given further insight into the complicated pathways involved in flowering time control in Medicago, while discovering some differences and similarities of the pathway to other plant species. Importantly, this work has highlighted key genes that require further investigation to uncover similar or unique characteristics of flowering time control in legume species.

## Data Availability

Sequence data is available at https://www.ncbi.nlm.nih.gov/bioproject/PRJNA794171 (accessed on 6 December 2021).

## Supplementary Materials

The following supporting information can be downloaded at: https://www.mdpi.com/article/10.3390/plants11030239/s1, Figure S1: Screening of a putative late-flowering TILLING population, Figure S2: SNP calling revealed a single point mutation in the *MtPφBS* gene, Figure S3: F2 population segregation phenotypes, Figure S4: The *MtphyA* and *MtphyB* mutants, Figure S5: Photo-morphogenic phenotypes for *Mtpφbs*, *MtphyA* and *MtphyB* mutants, Figure S6: Diurnal expression patterns for core clock genes in *Mtpφbs*, Figure S7: Circadian gene expression profiles of core clock genes in *Mtpφbs* mutant, Figure S8: Diurnal and Circadian Gene expression profiles of *MtPHYA* in *Mtpφbs* mutant, Table S1: Mapping Statistics for RNAseq of *Medicago truncatula* WT jester and *line#6*, Table S2: Flowering time candidate genes potentially affected in the *line#6*, Table S3: Genes containing SNP variants within 2MB of *MtPΦBS* gene in *line#6*, Table S4: List of primers used in this work.
