# Supplementary material for: A Point Mutation in Phytochromobilin synthase Alters the Circadian Clock and Photoperiodic Flowering of Medicago truncatula
Source: Plants (Basel). 2022 Jan 18;11(3):239. doi: 10.3390/plants11030239 (PMC8839385; doi:10.3390/plants11030239)
Supplement: Supplementary file 1 [file plants-11-00239-s001.zip › Supplementary_Figures_plants-1520812_17-01-21.pdf]

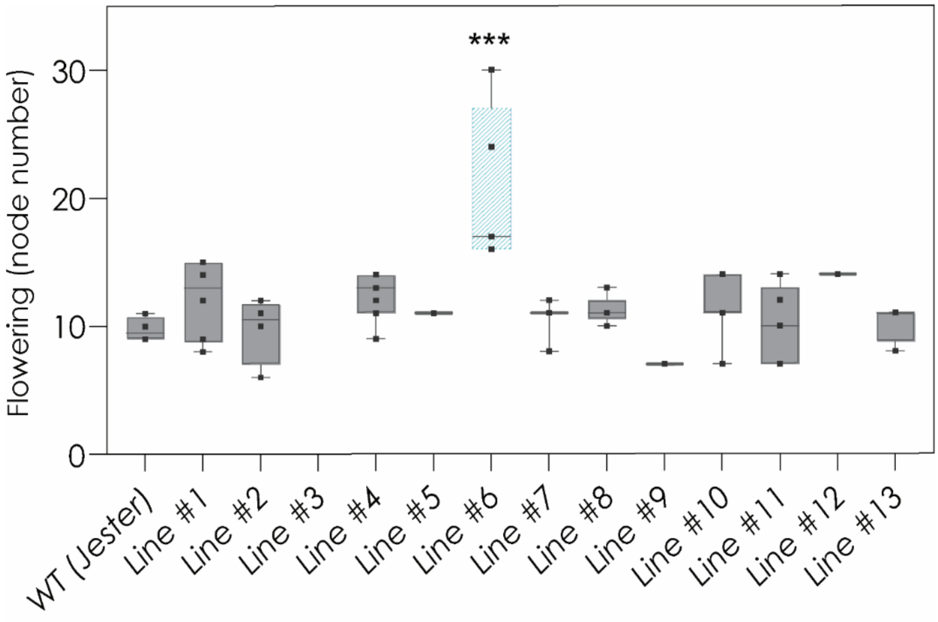

Supplemental Figure S1. Screening of a putative late-flowering TILLING population. Flowering time was measure in non-vernalized LD conditions (16 h of light/8 h of dark) as the number of nodes to first flower. *** p< 0.001 (*t-student*) line #6 vs WT(Jester).

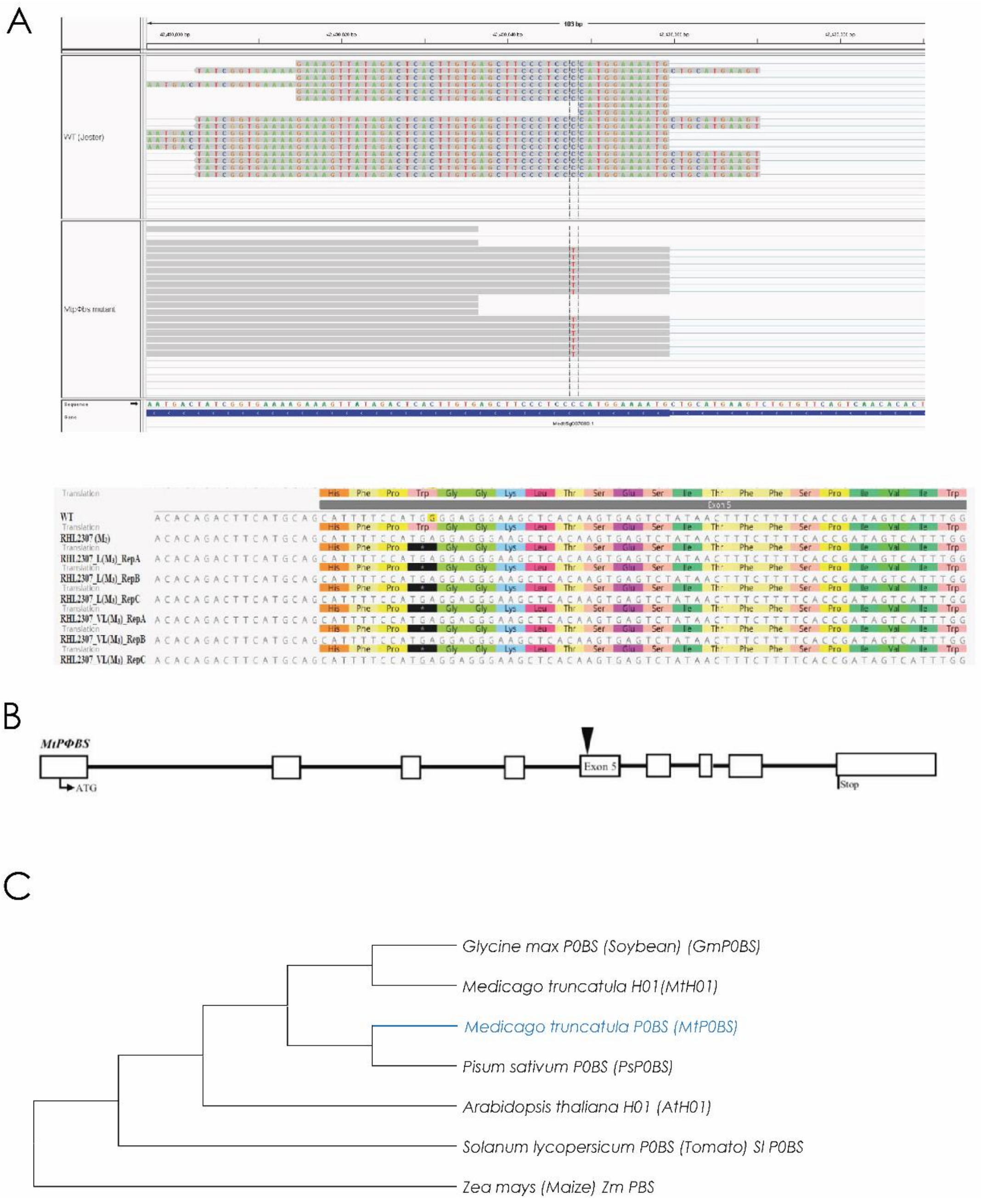

Supplemental Figure S2. SNP calling revealed a single point mutation in the *MtPφBS* gene. A) Screenshot of IGV program showing the aligned RNAseq reads for exon 5 of the MtPΦBS gene (Medtr5g097080) with mismatched nucleotide is shown in red in reverse strand (C to T). **B)** Exon 5 of the M*tPΦBS* gen showing that the G to A changed causes a change from a Tryptophan (Trp) to a premature stop codon. **C)** Evolutionary analysis by Maximum Likelihood method of *MtPΦBS* with *PΦBS* proteins from other plants species.

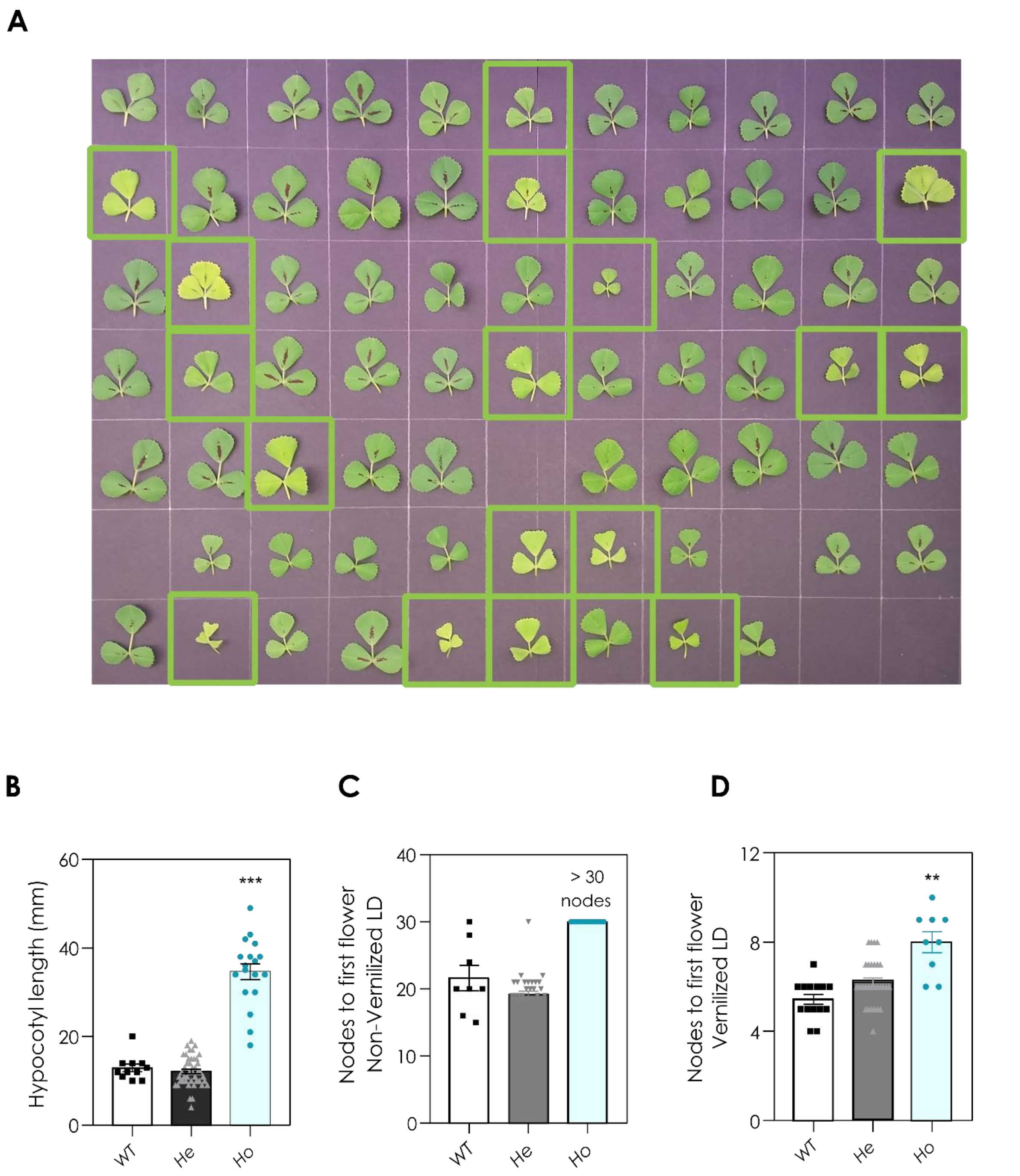

Supplemental Figure S3. F2 population segregation phenotypes. Seventy-five F2 plants grown under LD conditions. **A)** *Mtpφbs* mutants phenotype based on paler leave color**.** Picture of representative trifoliate leaves of the F2 segregating population. Missing plants died early in development. Green squares displays genotypic *Mtpφbs* mutation confirmed using the NCO1 assay. **B)** Length of hypocotyl at emergence of unifoliate (mm) of F2 segregating population. **C)** Flowering time measured in number of nodes to first flower under non vernilized LD conditions of F2 population **D)** Flowering time measured in number of nodes to first flower under vernalized LD conditions of F2 population. Ho= Homozygous *Mtpφbs* mutation, He = heterozygotes and WT = wild type genotype. *** p<0.001, ** p<0.01, (Student t test). Ho vs WT(Jester).

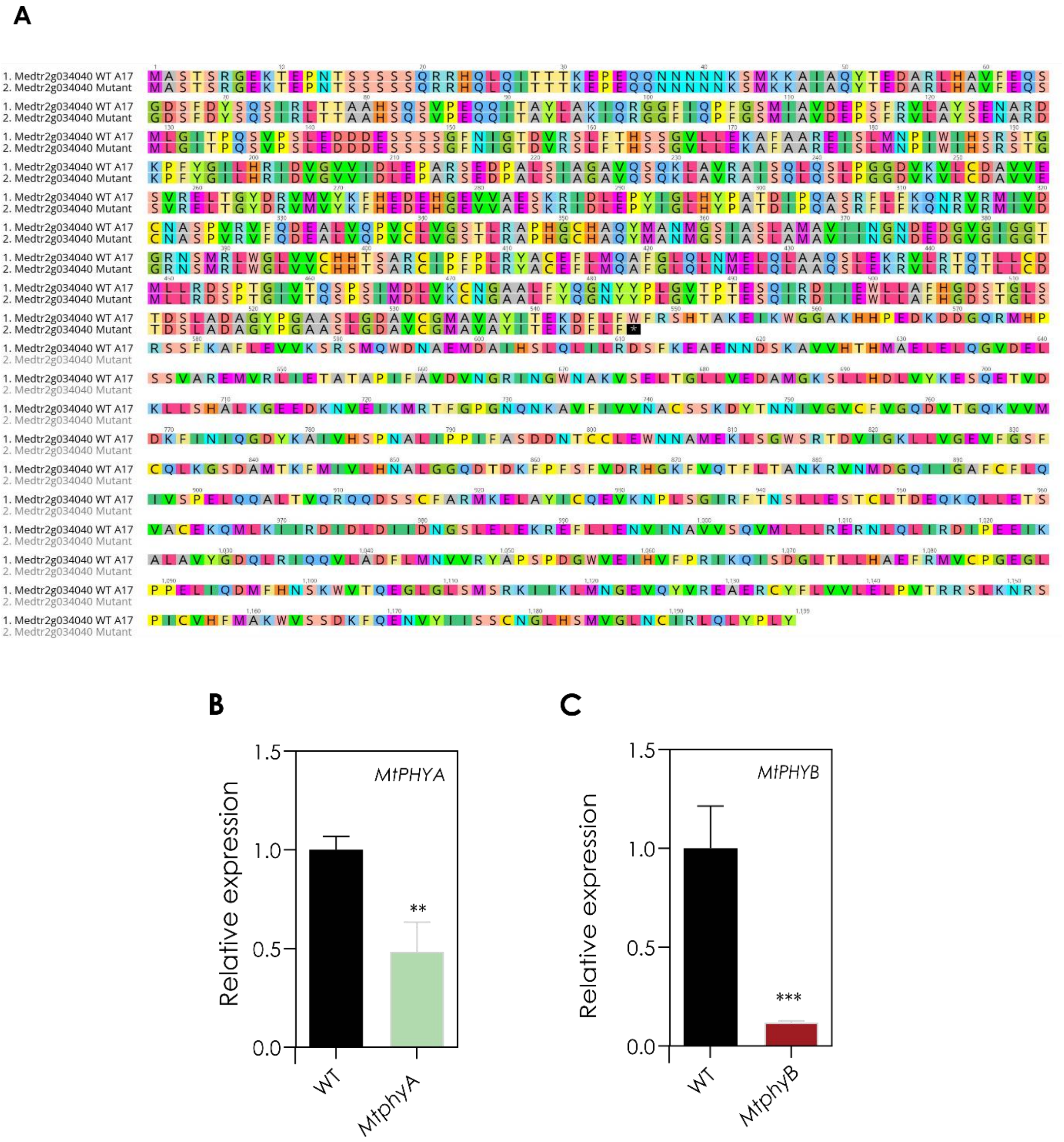

Supplemental Figure S4. The *MtphyA* and *MtphyB* mutants. A) Alignment of Medicago Medtr2g034040 (*MtPHYB*) protein sequence for A17 and *MtphyB* mutant, showing the changed at amionoacid triptofan 547 to a stop codon (Trp547>stop). Gene expression of the **B)** *MtPHYA* and **C)** *MtPHYB* genes in *Mtphya* and *MtphyB* mutants, and respective WT in LD. Relative transcript abundance was measured in the fully expanded trifoliate leaves of 14-day-old plants. Tissues were harvested 4h after dawn in LD conditions. Relative gene expression was measured by qRT-PCR with normalization to *MtPDF2*. Data are the mean ± SEM of three biological replicates and relative to the highest WT value. LD= long-day (16 h light/8 h dark). *** p<0.001, ** p<0.01, (Student t test).

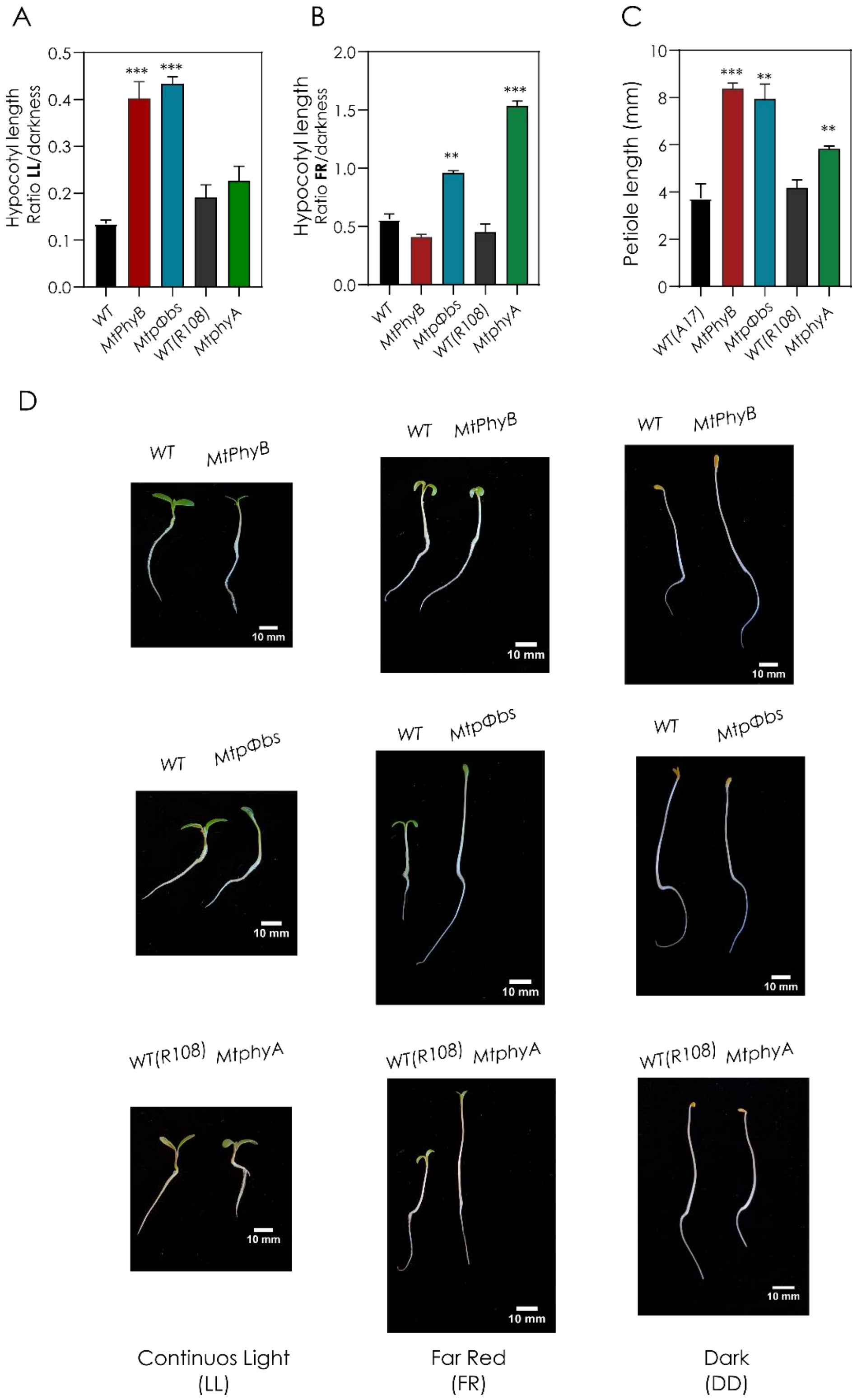

Supplemental Figure S5. Photo-morphogenic phenotypes for *Mtpφbs, MtphyA* and *MtphyB* mutants. Hypocotyl length relative to the dark control **A)** under continuous white light and **B)** under continuous FR light. **C)** Petiole length (mm) under LD conditions. **D)** Pictures of Hypocotyl at 7 days for each condition. WT (R108) is the background for *MyphyA* and WT(A17) for *MtphyB*. ***p < 0.001; **p<0.01, (Student t test). Error bars represent SEM. LD= long-day (16 h light/8 h dark). LL = continuous white light. FR = continuous FR light. Scale bar = 10mm.

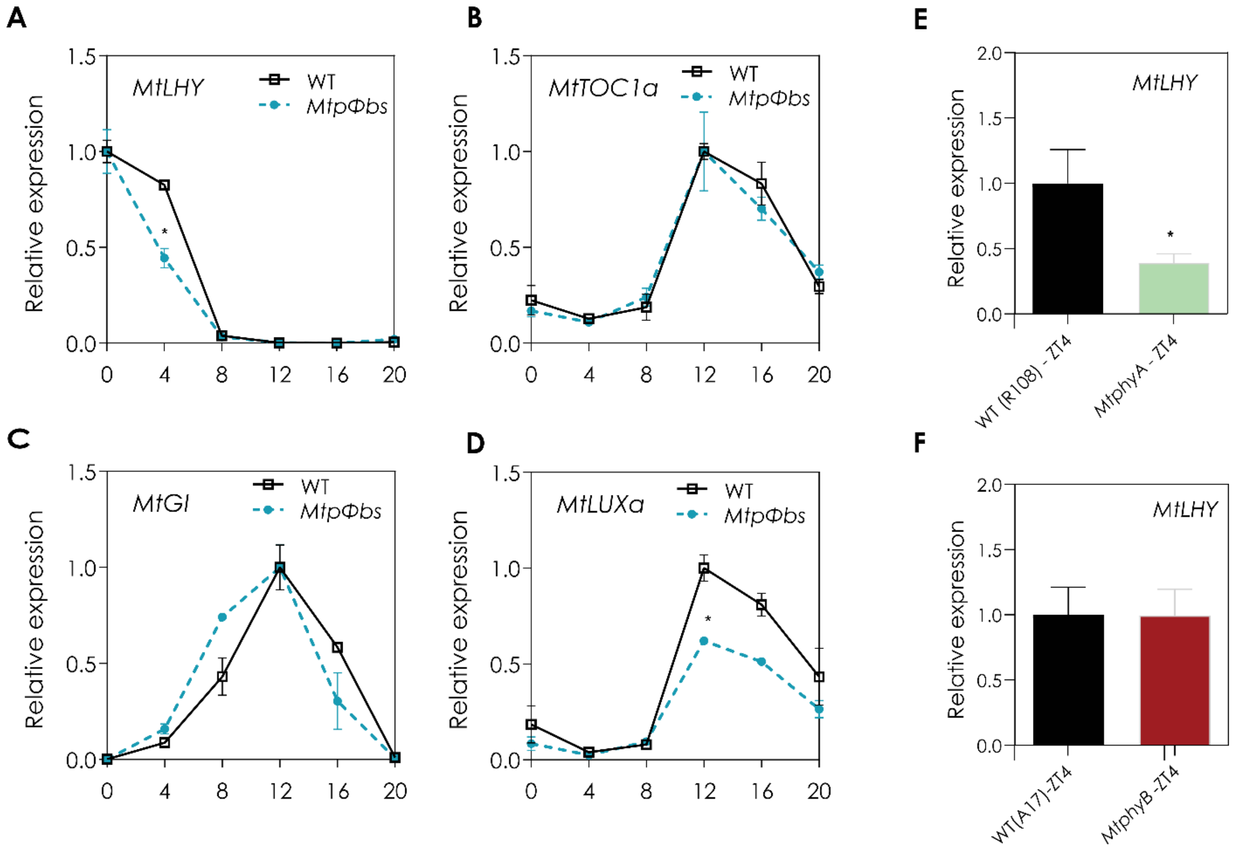

Supplementary Figure S6. Diurnal expression patterns for core clock genes in *Mtpφbs*
Expression profiles for ***A)*** *MtLHY*, ***B)*** *MtTOC1a*, ***C)*** *MtGI and* ***D)*** *MtLUXa.* Plants were entrained for 21 days under LD conditions. Tissues were harvested every 4h for 1 day. Relative expression of MtLHY in **E)** *MtphyA* and **F)** *MtphyB* of 21-day old plants harvested at 4h after dawn. Relative gene expression was measured by qRT-PCR with normalization to MtPDF2. Data are the mean ± SEM of three biological replicates and relative to the highest WT value. LD= long-day (16 h light/8 h dark). *p < 0.05 (Student t-test).

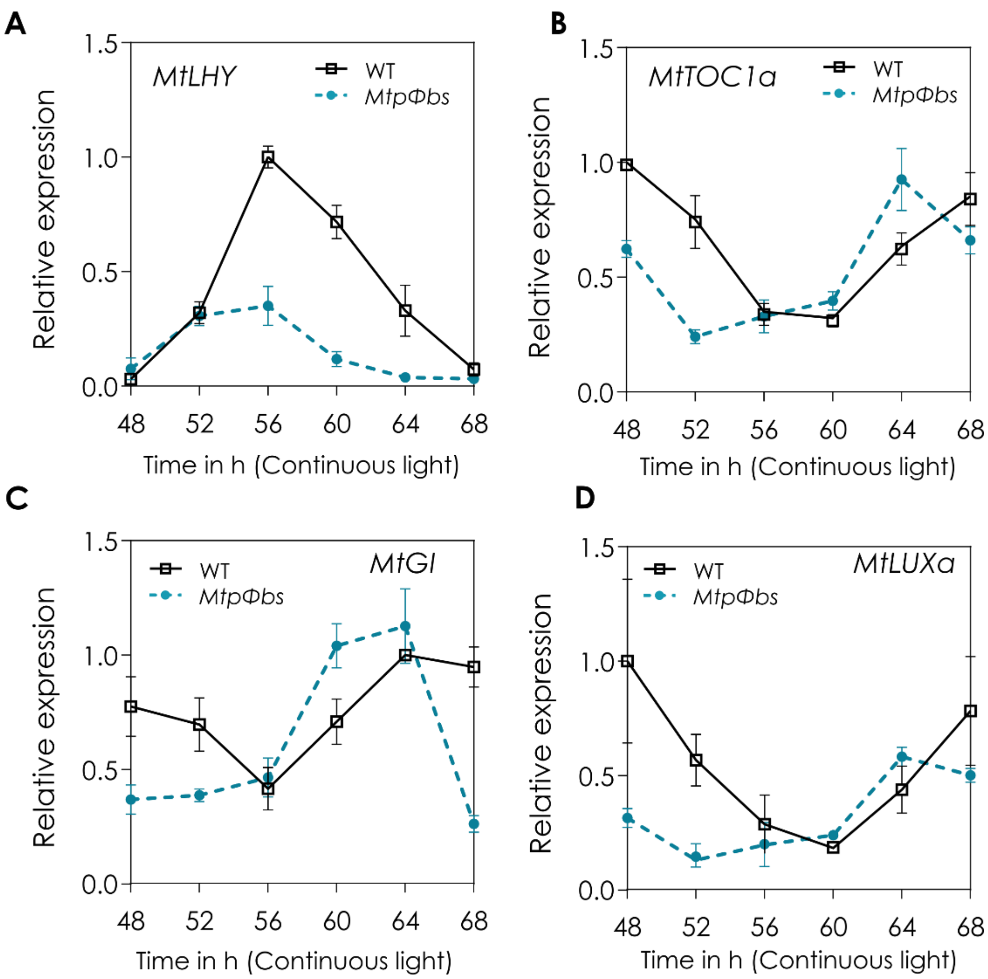

Supplementary Figure S7. Circadian gene expression profiles of core clock genes in *Mtpφbs* mutant. Expression profiles for ***A)*** *MtLHY*, ***B)*** *MtTOC1a*, ***C)*** *MtGI and* ***D)*** *MtLUXa.* Relative transcript abundance was measured in the fully expanded trifoliate leaves of 21-day-old plants. Tissues were harvested every 4h after dawn of the third day in continuous light after being entrained in LD for 18 days. Relative gene expression was measured by qRT-PCR with normalization to *MtPDF2*. Data are the mean ± SEM of three biological replicates and relative to the highest WT value. LD= long-day (16 h light/8 h dark).

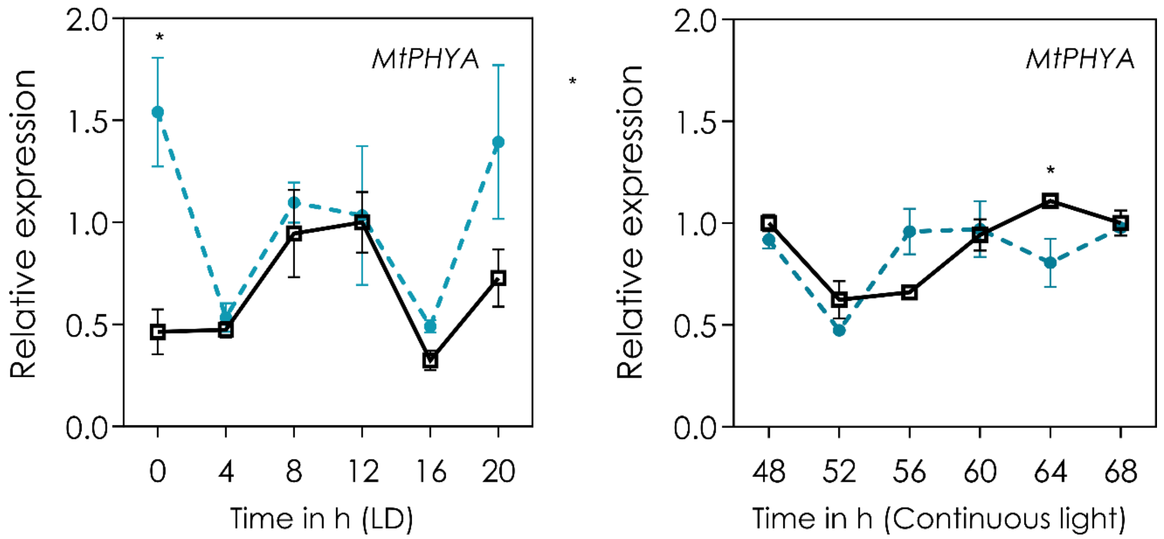

(A) (B)

Supplementary Figure S8. Diurnal and Circadian Gene expression profiles of *MtPHYA* *in Mtpφbs* mutant. Relative transcript abundance was measured in the fully expanded trifoliate leaves of 21-day-old *Mtpφbs* and WT plants in **A)** Diurnal (LD) and **B)** free-running (LL3) conditions. Tissues were harvested every 4h after dawn in LD conditions for 1 day (LD) and after three day in LL (LL3). Relative gene expression was measured by qRT-PCR with normalization to *MtPDF2*. Data are the mean ± SEM and relative to the highest WT value. LD= long-day (16 h light/8 h dark). LL3 = entrained in LD, moved to constant light and harvested third day. *$p < 0.05$ (Student t-test).
